# Unmet Needs for Dental Care Before and During the COVID-19 Pandemic in Greece: A Cross-Sectional Study

**DOI:** 10.3390/healthcare12222286

**Published:** 2024-11-15

**Authors:** Christos Ntais, Athina Charalampaki, Michael A. Talias, Nikolaos Kontodimopoulos, John Fanourgiakis

**Affiliations:** 1 Epidemiology Program, School of Science & Technology, Hellenic Open University, 26335 Patras, Greece; 2 Healthcare Management Program, School of Economics & Management, Open University of Cyprus, Nicosia 2220, Cyprus; 3 Healthcare Management Program, School of Social Sciences, Hellenic Open University, 26335 Patras, Greecenkontodi@hua.gr (N.K.); jfanourgiakis@hmu.gr (J.F.); 4 Department of Economics and Sustainable Development, Harokopio University, 17676 Athens, Greece; 5 Department of Management Science and Technology, Hellenic Mediterranean University, 72100 Agios Nikolaos, Greece

**Keywords:** unmet dental needs, COVID-19, European Union statistics on income and living conditions (EU-SILC), healthcare inequalities

## Abstract

Background/Objectives: Unmet dental needs involve cases wherein someone needed dental care and did not receive it. Published data on unmet dental needs are limited. This cross-sectional study investigates unmet dental needs in Greece before and during the COVID-19 pandemic. Methods: For this study, a questionnaire was created and distributed to a non-random sample. It was completed by 277 individuals. The questionnaire was based on the European Union Statistics on Income and Living Conditions (EU-SILC) tool, which is used to investigate unmet health needs. It was enriched with questions about unmet dental needs before and during the COVID-19 pandemic. Results: Of the 277 participants, 23.1% reported unmet dental needs before the onset of the COVID-19 pandemic, which decreased to 13.4% after the onset of the pandemic. However, a significant proportion of the sample (48.3%) reported no need for dental care after the onset of the COVID-19 pandemic. It is also worth noting that there were instances of dental visit avoidance, both when symptoms were present and for standard check-up/follow-up purposes, at rates of 17% and 27.8%, respectively. Conclusions: Unmet dental needs occurred at lower rates after the onset of the COVID-19 pandemic than those recorded before the pandemic. The main reasons for unmet needs before the pandemic were the cost of dental services and fear of treatment procedures or the visit to the dentist, while after the onset of the COVID-19 pandemic, lack of time and fear of coronavirus transmission were added.

## 1. Introduction

An unmet need for healthcare is defined as the difference between the necessary healthcare services and the services received [1,2,3]. It stems from barriers related to accessibility, availability and acceptability [4,5]. Similarly, unmet dental needs arise when an individual does not receive an available and effective dental treatment that could improve their oral health. Kim et al. [6] assessed unmet healthcare and dental needs from 2009 to 2022 using data from 2,750,212 individuals in Korea. Overall, the prevalence of unmet dental needs was significantly higher than that of unmet healthcare needs. A recent meta-analysis estimated a global prevalence of unmet dental needs in adolescents of 34%, with the highest prevalence found in Southeast Asia (72.3%) and the lowest in Europe (11.8%) [7]. Despite existing unmet dental needs, the importance of maintaining oral health is widely recognised. Agaku et al. [8] found that the mean number of school absence days because of illness was higher among students with an unmet therapeutic dental need than those reporting no unmet dental needs. Although most EU countries increasingly include dental coverage in healthcare insurance plans, recognising the importance of preventing oral diseases [9], more steps must be taken. Strategies to bridge this gap should focus on the financial aspect and on improving accessibility to dental services [10,11,12].

Several studies have identified inequalities in the distribution of dental care and unmet dental needs due to socio-economic barriers, geographic disparities, public health policies, cultural and knowledge barriers and social determinants of health [13,14,15,16]. Especially during the COVID-19 pandemic, unmet dental needs became a significant public health concern, as many people faced barriers to accessing essential dental services [15,16,17,18]. Lockdowns, clinic closures and infection control protocols limited routine dental visits, causing delays in preventive dental care and treatment. These disruptions disproportionately affected vulnerable populations, including low-income individuals, children and the elderly, who already faced challenges in obtaining regular dental care [19]. Karande et al. [14] examined unmet dental needs before and during the COVID-19 pandemic among U.S. children. Unmet dental needs increased from 2.9% in 2019 to 4.4% in 2020, and the highest prevalence of unmet dental needs was observed among disadvantaged segments of the population, including those who were uninsured, of low socio-economic status, living with their grandparents or living with a smoker. Ruff et al. [20] examined unmet dental needs in children following suspension of school-based oral health services due to COVID-19 in U.S. urban schools with a high proportion of low-income, minority students. They found that disruptions in school-based care due to COVID-19 pandemic control policies resulted in children losing access to their primary dental care provider.

Despite the high cost of dental services in Greece, the public healthcare system rarely provides coverage, resulting in out-of-pocket expenses. Especially during the recent economic crisis, such expenses were found to be catastrophic, and the level of oral health has deteriorated [13,21]. This cross-sectional study was conducted to investigate the trends in unmet dental needs before and after the onset of the COVID-19 pandemic in Greece and their causes. 

## 2. Methods

### 2.1. Research Tool

For the needs of this research, a questionnaire was created consisting of two parts (see Supplementary Materials). The first part contained questions related to the social and demographic characteristics of the respondents, such as gender, age, insurance status, annual personal income, educational level, marital status, number of children, etc. In addition, it included questions on self-assessment of oral health, the existence of dental problems affecting daily life (pain, reduced functionality, etc.) and the frequency of and reasons for visiting a dentist. The second part of the questionnaire contained questions regarding investigating unmet dental needs before and after the onset of the COVID-19 pandemic. These questions were based on the European Union Statistics on Income and Living Conditions (EU-SILC) tool used to investigate unmet healthcare needs, which was enriched with questions related to unmet dental needs before and during the pandemic. Dental needs were defined as the necessary dental care or treatment required to maintain or restore oral health, including preventive, restorative and emergency services. The questions on the unmet dental needs were divided into two different periods: before the onset of the pandemic (covering the twelve months from March 2019 to February 2020) and after the onset of the pandemic (March 2020 to December 2020).

### 2.2. Data Collection

This research collected data by distributing the questionnaire to a non-random sample of the general population, either electronically (via Google Forms) or in hard copy, using the snowball method. First, a small group of initial participants within the general population was identified. Each initial participant was then asked to refer others they knew. Referred participants were then asked to refer additional contacts. This referral-based chain continued, with each wave bringing in new participants who referred additional contacts. An important aspect of this approach is that no distinction was made between people with dental needs and those without. The questionnaires were distributed and completed from November 2021 to February 2022. Overall, 292 questionnaires were answered, 277 of which were fully completed and analyzed.

### 2.3. Code of Ethics

This study was conducted in compliance with the Declaration of Helsinki and approved by the competent body of the Postgraduate Program of Studies “Healthcare Management” of the Hellenic Open University (Ref. No 151134/29 October 2021). All participants’ written informed consent was secured before their inclusion in the study.

### 2.4. Statistical Analysis

The mean and standard deviation were used to describe the quantitative variables. In contrast, the median and interquartile range were used to describe the quantitative variables that did not follow a normal distribution. Absolute (N) and relative (%) frequencies were used to describe qualitative variables. The Mann-Whitney non-parametric test was used to compare quantitative variables between two groups. The Bonferroni correction was used to control for type I error due to multiple comparisons, according to which the significance level is 0.05/κ (κ = number of comparisons). The Chi-squared test was used to determine if two qualitative variables were related, while Fisher’s Exact test was used in cases where the conditions for applying the above test were not met. The McNemar test was used to compare the frequency of participants’ visits to the dentist and their unmet dental needs before and after the onset of the COVID-19 pandemic. Multivariate logistic regression analysis was performed to identify factors significantly associated with the likelihood that participants needed dental care and did not receive it before and after the onset of the pandemic. Significance levels were two-sided, and statistical significance was set at 0.05. The statistical program SPSS v22.0 was used.

## 3. Results

### 3.1. Demographics and Social Characteristics

The study sample comprised 277 subjects. Demographic and social characteristics of the sample are presented in Table 1.

### 3.2. Preference of Dental Service Provider

Regarding the choice of dental service provider, 231 (83.4%) respondents stated that they chose a “private dental office”, 38 (13.7%) a “dental office operating within a hospital outpatient’s clinic” and 8 (2.9%) a “dental office of a healthcare centre”.

### 3.3. Self-Assessment of Oral Health

Regarding the self-assessment of their oral health, 15 (5.4%) respondents answered “poor”, 83 (30%) “fair”, 126 (45.5%) “good” and 53 (19.1%) “very good” or “excellent”. To the question, “Are there any dental/oral problems that affect your daily life?”, 66 (23.8%) respondents answered in the affirmative.

### 3.4. Frequency and Reason for Visiting a Dentist Before the COVID-19 Pandemic

Regarding the frequency and reason for visiting a dentist before the onset of the COVID-19 pandemic, “less than once a year” was answered by 57 (20.6%), once a year by 124 (44.8%), twice a year by 76 (27.4%) and “more than twice a year” by 20 (7.2%) respondents.

Regarding the reason for visiting a dentist, “therapeutic reasons (fillings, endodontic treatments, prosthetic restorations, etc.)” were indicated by 130 (46.9%), “aesthetic interventions/restorations” by 9 (3.2%), “standard check-up/follow-up” by 133 (48%) and “dental cleaning” by 5 (1.8%) respondents.

### 3.5. Unmet Needs for Dental Services

All identified cases of unmet dental needs fall into one of the following categories: therapeutic needs (fillings, endodontic treatments, prosthetic restorations, etc.), aesthetic interventions/restorations, standard check-ups/follow-ups and dental cleaning.

#### 3.5.1. Before the Onset of the COVID-19 Pandemic

Unmet needs for dental services before the onset of the COVID-19 pandemic were reported by 64 (23.1%) respondents. Regarding the reasons, “high cost of examination/treatment” was reported by 29 (46%), “waiting time for visit/treatment at the dentist’s was too long” by 11 (17.5%), “there was no time available on my part” by 9 (14.3%), “distance/difficulty in accessing—travelling” by 6 (9.5%), “fear of the dental visit/examination/treatment” by 14 (22.2%), “I wanted to wait and see if the problem would go away on its own” by 12 (19%), “I don’t know a good dentist” by 4 (6.3%) and “other” by 4 (6.3%) respondents. It is worth noting that before the pandemic, 11.2% of the respondents expressed some concern about transmitting diseases during their visits to the dentist.

To identify the factors that were significantly associated with the likelihood that participants did not receive dental care when they needed it, a multivariate logistic regression analysis was performed, with independent variables being the participants’ demographic characteristics, their responses regarding their oral health and whether they had a concern about disease transmission during their dental visit. According to the regression results, it appears that the participant’s place of residence, the concern related to the transmission of diseases during their dental visit, dental or oral problems that affected their daily life and their oral health assessment influenced the likelihood that the participants did not receive dental care when they needed it. More specifically, participants who resided outside of Attica were 2.12 times more likely to have not received dental care while in need compared to participants who lived in Attica. In addition, those who had a concern about disease transmission before the onset of the pandemic when visiting the dentist were 2.76 times more likely to have not received the necessary dental care compared to participants who did not have such a concern. Participants with dental or oral problems that affected their daily lives showed a higher probability of not receiving dental care while in need than participants who did not face such issues. Finally, the better participants rated their oral health, the less likely they were to have not received dental care while in need.

#### 3.5.2. After the Onset of the COVID-19 Pandemic

Unmet needs for dental services during the COVID-19 pandemic were reported by 37 (13.4%) respondents. Also, 17%, even when symptomatic, avoided visiting their dentist, and 27.8% avoided visiting their dentist for standard check-ups/follow-ups. Of those who did not visit the dentist after the onset of the pandemic, 48.3% said there was no need, while 31.7% cited financial reasons and 21.7% cited concern regarding the spread of COVID-19. After vaccinations started in December 2020, 74% felt extremely safe visiting their dentist and 91.7% believed that stricter protocols of cleanliness and hygiene in the dental office were followed after the onset of the pandemic.

To identify factors that were significantly associated with the likelihood that participants did not receive dental care when they needed it, a multivariate logistic regression analysis was performed, with the independent variables being the participants’ demographic characteristics, their responses regarding their oral health, whether they had a concern about disease transmission when visiting the dentist before the pandemic started and how safe they felt visiting the dentist. Participants who lived outside the region of Attica were 3.68 times more likely to have not received dental care while in need of it compared to participants who lived in Attica. Participants who had children (compared to those who did not) were 2.18 times more likely to have not received dental care while in need. In addition, those who, prior to the onset of the pandemic, had a concern about disease transmission when visiting the dentist were 3.26 times more likely to have not received necessary dental care compared to participants who did not have such a concern. Participants who had dental or oral problems that affected their daily lives were 4.86 times more likely to have not received dental care when they needed it than participants who did not have such problems. In addition, those who believed that stricter hygiene and cleanliness protocols were followed in the dental office in view of the pandemic were 70% more likely to have received the necessary dental care compared to participants who did not believe that such protocols were followed. Finally, the better participants rated their oral health, the more likely they were to receive dental care when needed.

### 3.6. Comparison of Unmet Dental Needs Before and During the COVID-19 Pandemic

As shown in Table 2, which presents the proportion of participants who did not receive dental care when needed before and after the onset of the COVID-19 pandemic, there was a significant reduction in unmet dental needs after the onset of the pandemic.

At the same time, as shown in Table 3, which presents the proportion of participants in terms of the frequency of visits to the dentist before and after the onset of the COVID-19 pandemic, there was a significant decrease in the frequency of visits to the dentist after the onset of the pandemic.

More specifically, the proportion of participants who visited the dentist less than once a year before the pandemic increased after the pandemic started, while the proportion of participants who visited once a year decreased. In addition, the proportion of participants who visited the dentist twice a year before the pandemic decreased after the pandemic started, while the proportion of participants who visited their dentist more than twice a year increased. Finally, it is worth noting that the proportion of participants who visited their dentist at least twice a year before the pandemic (34.7%) decreased significantly (*p* = 0.043) compared to the proportion during the pandemic (28.5%).

## 4. Discussion

The present study assessed unmet dental needs before and during the COVID-19 pandemic in Greece. Of the total 277 respondents, 64 (23.1%) reported unmet dental needs before the pandemic started. The main reasons were the cost of dental services, concern about the dental visit and related treatment procedures, the expectation that the problem would subside without intervention, long waiting times, lack of time on the part of patients and, finally, difficulty in access. During the pandemic, out of a total of 277 respondents, 37 (13.4%) reported an unmet need for dental services. The main reasons were cost, concern about the spread of COVID-19, lack of time, belief that the problem would go away on its own and difficulty in travelling. These results align with similar studies conducted in the past [7,22,23], which showed financial and access reasons as the most important ones for unmet healthcare needs. However, it is worth mentioning that no data are available specifically for dental needs, both before and after the onset of the COVID-19 pandemic.

Comparing the rates of unmet dental needs before and during the COVID-19 pandemic, it was evident that the rate decreased after the pandemic started. This finding can be attributed to the fact that a large proportion of participants (48.3%) did not experience a need for dental care after the onset of the pandemic. Of course, the respondents made this assessment, so it is a subjective rather than objective fact. It should also be noted that while 13.4% reported unmet dental needs after the pandemic started, a significant proportion avoided visits to the dentist, even when symptomatic (17%) and for standard check-ups/follow-ups (27.8%). In addition, considering the period of the pandemic for which the participants were interviewed, i.e., from March 2020 to December 2020, it is evident that the 12-month period for which unmet healthcare needs are usually assessed is incomplete. Therefore, the period under consideration may not have been long enough for a need for dental care to arise.

Examining the variables that seemed to influence unmet dental needs, it was found that the place of residence was one of them. Specifically, participants residing in a rural area were 2.12 times more likely to report unmet dental needs, which increased to 3.68 after the pandemic started. This increase can be attributed to the ban on inter-county and county-to-county travel implemented during the first months of the COVID-19 pandemic, as it is common for people living in provincial and/or island areas of Greece to visit a dentist located in a larger urban or semi-urban centre. Also, having children seemed to be associated with cases of unmet dental needs after the onset of the pandemic. Specifically, compared to those who did not, participants with children were 2.18 times more likely to have not visited their dentist while in need. This finding can be attributed to the fact that during the months of the pandemic for which participants were interviewed, schools and extracurricular activities were suspended. Therefore, children attended their lessons at home via the Internet while abstaining from extracurricular activities. As a result, parents were required to have children under their supervision for almost the entire day, limiting their flexibility for outside activities.

The concern about disease transmission in the dental office also influenced the likelihood of unmet dental needs. Those who reported a related concern before the pandemic were 2.76 times more likely to avoid visiting their dentist. This likelihood increased after the pandemic started and reached 3.26 times. However, most respondents reported that increased hygiene measures in the dental office provided an extra sense of security regarding the spread of COVID-19. Specifically, it was found that those who believed that stricter protocols of cleanliness and hygiene were followed were 70% more likely to visit their dentist. The relationship between the concern about coronavirus transmission and the likelihood of unmet dental needs agrees with the results of Kim et al. [24], where patients who reported fear of COVID-19 transmission made fewer medical visits during the early period of the pandemic.

Significant differences were found in the frequency of visits to the dentist before and after the onset of the pandemic. It has been established that the ideal interval for visits to the dentist is every 6 to 12 months [25]. In our study, 34.7% of respondents reported visiting the dentist at least twice a year before the COVID-19 pandemic. This percentage has decreased to 28.5% since the onset of the pandemic. This decrease can be attributed both to hesitancy due to concern about coronavirus spread, to the travel ban during the early months of the pandemic and to the lack of time reported by a significant proportion of participants.

To the best of our knowledge, this is the first study ever carried out in Greece assessing unmet dental needs before and after the onset of the COVID-19 pandemic. As its sample included a range of subjects aged from 18 to over 65 years old, it addresses a broad range of dental needs that could arise across the long timeframe covered. The study’s focus on unmet dental needs provides valuable insights into accessibility trends and informs healthcare policies aimed at improving dental care equity. However, the study also has limitations. The reliance on self-reported data could introduce recall bias, especially regarding participants’ recollections of pre-pandemic dental care experiences. On the other hand, without longitudinal follow-up, it is challenging to determine whether observed changes in unmet dental needs reflect lasting improvements or temporary adaptations.

Future research directions could focus on examining the specific factors that contribute to individuals avoiding dental visits, even when symptomatic or for routine check-ups. Future research would provide insights into whether the reduction in unmet dental needs observed post-pandemic represents a sustained improvement or a temporary shift. Further studies could also explore demographic-specific trends, identifying whether certain populations continue to face barriers to dental care. Investigating the impact of remote consultations or alternative models of dental care, which gained traction during the COVID-19 pandemic, may shed light on innovative solutions to reduce unmet dental needs. Additionally, qualitative studies capturing patients’ perspectives on their dental care experiences post-pandemic would add depth to quantitative findings, providing a more comprehensive understanding of remaining challenges and effective strategies to address them. This multi-faceted approach could guide policies and interventions aimed at further enhancing dental care accessibility and equity.

## 5. Conclusions

The present study uniquely assesses the unmet dental needs in Greece before and during the onset of the COVID-19 pandemic. The contributing factors are usually included in those of unmet healthcare needs as a whole, without presenting independent data specific to dental care. The study’s findings indicate a promising reduction in unmet dental care needs since the onset of the COVID-19 pandemic, suggesting improvements in accessibility or adaptation of dental services. However, the data reveal a need for further investigation into patterns of dental care avoidance, particularly among individuals who refrained from seeking care despite the presence of symptoms or for routine check-ups and follow-ups. Understanding the factors that contribute to this avoidance—whether due to concerns related to the pandemic, financial challenges or shifts in health prioritisation—will be critical for addressing remaining barriers to dental care access. Further research should focus on these dynamics to ensure sustained progress in meeting dental needs and to support targeted strategies that encourage timely and preventive dental visits across all segments of the population.

The COVID-19 pandemic highlighted the critical role of dental care in overall health and underscored the need for resilient healthcare systems that ensure access to dental services even during public health crises. The issue of unmet dental needs and how they are shaped after the onset of a pandemic is an area that needs intensive future research, as the possibility of new pandemics in the near future is a reality. The results of such research will be useful tools for policy makers in the health and dental care sector and for those who attempt to further investigate the issue of unmet healthcare needs as a whole.

## Figures and Tables

**Table 1:** Demographic and social characteristics of the sample (N = 277).

		N	%
Gender	Male	103	37.2
	Female	171	61.7
	Other	3	1.1
Age group	18–24	40	14.4
	25–34	84	30.3
	35–44	70	25.3
	45–54	39	14.1
	55–64	29	10.5
	65+	15	5.4
Educational level	Primary school	3	1.1
	Junior high school	10	3.6
	High school	74	26.7
	University	136	49.1
	Postgraduate/doctoral degree	54	19.5
Employment	Civil servant	68	24.5
	Private employee	94	33.9
	Freelancer	33	11.9
	Retired	27	9.7
	Pupil/Student	32	11.6
	Unemployed	23	8.3
Place of residence	Within the Region of Attica	186	67.4
	Outside the Region of Attica	90	32.6
Family situation	Married	112	40.4
	Unmarried	138	49.8
	Divorced	14	5.1
	Widow/er	11	4.0
	With civil partner	2	0.7
Child/children	Yes	118	42.6
	No	159	57.4
Annual Personal Income (Euro)	0–5000	74	26.7
	5000–10,000	39	14.1
	10,000–15,000	94	33.9
	15000–20,000	33	11.9
	20,000–25,000	15	5.4
	25,000–30,000	10	3.6
	30,000+	12	4.3
Insurance status	Uninsured	16	5.8
	Insured with a public institution	188	67.9
	Insured with a private institution	73	26.4

**Table 2:** Comparison of unmet dental needs before and after the onset of the COVID-19 pandemic.

		Before the Onset of the Pandemic		After the Onset of the Pandemic		*p*-Value
		N	%	N	%	
Has there ever been a time when you needed dental care and did not receive it?	No	213	76.9	240	86.6	<0.001
	Yes	64	23.1	37	13.4	

**Table 3:** Frequency of visits to the dentist before and after the onset of the COVID-19 pandemic.

		Before the Onset of the Pandemic		After the Onset of the Pandemic		*p*-Value
		N	%	N	%	
How frequent were your visits to the dentist?	Less than once a year	57	20.6	82	29.6	0.005
	Once a year	124	44.8	116	41.9	
	Twice a year	76	27.4	53	19.1	
	More than twice a year	20	7.2	26	9.4	

## Data Availability

The data that support the findings of this study are available from the corresponding author upon reasonable request.

## Supplementary Materials

The following supporting information can be downloaded at: https://www.mdpi.com/article/10.3390/healthcare12222286/s1, Annex: Questionnaire to investigate unmet dental needs before and after the onset of the COVID-19 pandemic in Greece.
