# Supplementary material for: Unmet Needs for Dental Care Before and During the COVID-19 Pandemic in Greece: A Cross-Sectional Study
Source: Healthcare (Basel). 2024 Nov 15;12(22):2286. doi: 10.3390/healthcare12222286 (PMC11593393; doi:10.3390/healthcare12222286)
Supplement: Supplementary file 1 [file healthcare-12-02286-s001.zip › healthcare-3272406-supplementary.pdf]

Annex: Questionnaire to investigate unmet dental needs before and after the onset of the COVID-19 pandemic in Greece

According to the Organisation for Economic Co-operation and Development (OECD), ***unmet need for health (or dental) care is defined as cases where, while there was a need for a medical/dental examination or treatment, the person did not receive it.*** The following questionnaire, which you are invited to complete, consists of **27 short questions** and aims to investigate the existence of unmet need for dental care and whether and how it has been affected by the COVID-19 pandemic. The survey is being conducted as part of the **Master's Degree Program "Health Care Management" at the Hellenic Open University.**

The questionnaire is ***anonymous*** and completed ***voluntarily***. The results will be used exclusively for research purposes. After carefully studying the statements, please answer honestly and as accurately as possible.

If you agree to participate in this survey, please read the following statements:

"I confirm that I have read and understood the above information for this study. I understand that my data will be anonymous and that my participation is voluntary. I understand that I have the right to withdraw from the study at any time and I agree to allow the data collected to be used for this research. I agree to the above statements and I am willing to participate in this study".

☐ I consent

☐ I do not consent

Part I - Demographics

1. **Gender:**

☐ Male

☐ Female

☐ Other

2. **Age:**

☐ 18-24

☐ 25-34

☐ 35-44

☐ 45-54

☐ 55-64

☐ 65+

3. **Educational level:**

☐ Primary school

☐ Junior high school

☐ Senior high school

☐ University

☐ Postgraduate/doctoral degree

4. **Type of employment:**

☐ Civil servant

☐ Private Employee

☐ Freelancer

☐ Retired

☐ Pupil / Student

☐ Unemployed

5. **Place of residence (county, city):**

6. **Family situation:**

☐ Married

☐ Unmarried

☐ Divorced

☐ Widow/er

☐ With civil partner

7. **How many children you have:**

☐ 0

☐ 1

☐ 2

☐ 3

☐ 4

☐ 5

☐ Other:.............

8. **Annual personal income (in euro):**

☐ 0 - 5,000

☐ 5,000 - 10,000

☐ 10,000 - 15,000

☐ 15,000 - 20,000

☐ 20,000 - 25,000

☐ 25,000 - 30,000

☐ Above 30,000

9. **Insurance Status:**

☐ Uninsured

☐ Insured with a public institution

☐ Insured with a private institution

Part II - Questions regarding unmet dental needs

In order to facilitate the completion of the questionnaire, it is specified that the questions relate to ***two different time periods****, which are defined as follows:*

- ***Period before the onset of the pandemic****: March 2019 to February 2020*
- ***Period after the onset of the pandemic****: March 2020 to December 2020*

10. **Dental service provider:**

☐ Private dental office

☐ Dental office operating within a hospital outpatient's clinic

☐ Dental office of a healthcare centre

☐ Other : ....

11. How would you rate your oral health?

☐ 1 (Poor)

☐ 2 (Fair)

☐ 3 (Good)

☐ 4 (Very good)

☐ 5 (Excellent)

12. Are there any dental/oral problems that affect your daily life?

☐ Yes

☐ No

13. Before the onset of the pandemic, how often did you visit your dentist?

☐ Less than once a year

☐ Once a year

☐ Twice a year

☐ More than twice a year

14. What was the most common reason for your visit to the dentist?

☐ Therapeutic reasons (fillings, endodontic treatment, prosthetic restorations, etc.)

☐ Aesthetic interventions / restorations

☐ Standard check-up/follow-up

☐ Other: ....

15. Has there ever been a time in the period March 2019 - February 2020 when you needed dental care (for diagnostic or therapeutic reasons) but did not receive it?

☐ Yes

☐ No

If you answered ***yes*** *to the above question, please specify the reasons* ***(up to two)*** *based on the options below:*

☐ High cost of examination/treatment

☐ Waiting time for visit/treatment at the dentist's was too long

☐ There was no time available on my part

☐ Distance / difficulty in accessing - travelling

☐ Fear of the dental visit / examination / treatment

☐ I wanted to wait and see if the problem would go away on its own

☐ I don't know a good dentist

☐ Other *(specify):* ....

16. **Before the pandemic started, did you have any concern about disease transmission during your visits to the dentist?**

☐Yes

☐No

17. **Since the start of the pandemic, have you visited your dentist?**

☐ Yes

☐ No

18. **If you answered yes to the above question, what are the main reasons for visiting the dentist since the onset of the pandemic?**

☐ Therapeutic reasons (fillings, endodontic treatment, prosthetic restorations, etc.)

☐ Aesthetic interventions / restorations

☐ Standard check-up/follow-up

☐ Other: ....

19. **If you haven't visited your dentist since the pandemic started, what prevented you? (choose up to two reasons from the following)**

☐ Financial reasons

☐ I couldn't find an available appointment

☐ I didn't have time

☐ Distance / Difficulty in accessing - travelling

☐ Fear of the dental visit / examination / treatment

☐ I wanted to wait and see if the problem would go away on its own

☐ I don’t know a good dentist

☐ Concern about COVID-19 transmission

☐ Other: ....

20. **What was the frequency of your visits to the dentist since the onset of the pandemic?**

☐ Less than once a year

☐ Once a year

☐ Twice a year

☐ More than twice a year

21. **Since the onset of the pandemic has there been any occasion where, while you needed dental care, you did not receive it?**

☐ Yes

☐ No

22. **After the onset of the pandemic, has there been any occasion when, while there were symptoms (e.g. pain, swelling, etc.) you avoided visiting your dentist?**

☐ Yes

☐ No

23. **Since the onset of the pandemic, has there been any occasion when you have avoided visiting your dentist for a check-up or follow-up?**

☐ Yes

☐ No

24. Regarding the risk of spreading COVID-19, after the start of vaccinations in December 2020, what is the level of safety you feel to visit your dentist?

☐ None

☐ Low

☐ Moderate

☐ High

☐ Extremely high

25. In view of the pandemic, do you think stricter protocols of cleanliness and hygiene are being followed in the dental offices?

☐ Yes

☐ No

26. If you answered yes to the above question, has this affected the level of safety you feel in the dental office regarding the transmission of COVID-19 or other diseases?

☐ Not at all

☐ A little

☐ Moderately

☐ Much

☐ Very much
